# Status and Influencing Factors of Nurses' Perception of Toxic Leadership Behavior: A Cross-Sectional Study

**DOI:** 10.1155/2023/7711237

**Published:** 2023-11-29

**Authors:** Xueqin Guo, Xin Li, Yuhan Wang, Yumei Wang, Huan Jin, Fang Xiao, Yuting Xiang, Chenzi Xu, Yangjing Wang, Jia He, Lijuan Xiong

**Affiliations:** ^1^Union Hospital, Tongji Medical College, Huazhong University of Science and Technology, Wuhan, Hubei 430022, China; ^2^School of Nursing, Tongji Medical College, Huazhong University of Science and Technology, Wuhan, Hubei 430030, China

## Abstract

**Aim:**

The aim of this study is to analyse the toxic leadership behavior of nurse managers perceived by nurses and its related factors.

**Background:**

Toxic leadership is becoming more common as a risk factor in nursing. However, there is a scarcity of research on the elements that influence toxic leadership practices from the perspective of nurses' perceptions.

**Methods:**

A cross-sectional study was conducted with 455 nurses from August to October 2022. A demographic information questionnaire and a negative behavior scale for nurse managers were used. Descriptive statistics, Kruskal–Wallis H test or Mann–Whitney *U* test, and multiple linear regression were used to explore the relevant factors of nurses' perceived toxic leadership behaviors of nurse managers.

**Results:**

The population was dominated by 423 (92.97%) females, 318 (69.89%) married, and 420 (92.31%) with a bachelor's degree. The toxic leadership behavior scale score for nurse managers was 109 (87, 123) and the score for each entry was (2.94 ± 0.92). Gender, educational level, department, number of night shifts, and nature of employment were the influencing factors of the negative leadership behavior of nurse leaders as perceived by nurses (*P* < 0.05), explaining a total of 43.1% of the total variance.

**Conclusion:**

In general, nurses' perceived toxic leadership behaviors of nurse managers were at a moderate level. More toxic leadership behaviors were observed by nurses who were female, less educated, on busy units, with unstable nature of appointments, and with frequent night shift rotation. *Implications for Nursing Management*. Focus on the psychological condition of nurses who are female, less educated, work in busy units, have an unstable nature of employment, and rotate night shifts frequently. The negative impacts of toxic leadership behaviors might be lessened as a result.

## 1. Background

Nurse managers are critical to the efficient development of the nursing workforce, the maintenance of nurses' physical and emotional well-being, and the dynamic and efficient operation of nursing care [1]. High expectations and demands simultaneously determine the difficulties and challenges of the nurse manager profession [2]. Effective nurse managers can establish a healthy work environment, ensure patient safety, and facilitate the achievement of organizational goals [3]. It is important to emphasize, however, that not all nurse managers possess effective leadership abilities; there are indeed negative and ineffective leadership behaviors—toxic leadership behavior [4–7].

Toxic leadership behavior in nurse managers, as the dark side of leadership conduct, has been shown to have numerous negative repercussions on nurses, organizations, and patients. For nurses, it reduces job satisfaction and productivity [5], devalues professional worth and belief cognition [8], puts them at high-stress levels, resulting in absenteeism and high turnover intention [9], and makes them more likely to retaliate [10]. Although nurses may purposefully avoid toxic management, it could eventually lead to greater exhaustion due to the inability to obtain important information or depletion of remaining resources [11]. For patients, nurses report more adverse events, nursing quality is poor [12, 13], and patient satisfaction decreases [14]. For organizations, it may put the nursing profession in a difficult position since it has been shown to negatively impact organizational performance [6], cause team relationship conflicts [15], generate a toxic organizational culture, and even legitimize harmful leadership behavior [16]. The negative impact is often greater than the positive impact; even the actual or potential dangers brought by low-level toxic leadership cannot be ignored. The prevention and avoidance of toxic leadership behavior are more important than the cultivation and promotion of positive leadership [17].

When it comes to the factors that promote the generation of the toxic leadership behavior, the toxic triangle [18] considers that toxic leadership is an integration of subordinate performance, leadership characteristics, and organizational environment during work interaction. Existing studies on the reasons of toxic leadership behavior by nurse managers focus on the nurse manager and organizational aspects, whereas nurses' personal opinions need to be expanded. According to Estes [19], nurse managers' feelings of organizational injustice, autocratic personalities, severe workload, and lack of management experience were all variables that contributed to toxic leadership. High-self-esteem [20] managers are more prone to develop a compulsive work passion that eventually causes self-exhaustion and more toxic leadership perceived by their subordinates. Nurse managers who work part-time, have little management experience, and have heavy management tasks lead to more toxic leadership. Naturally, the level and type of hospital where the nurse manager works also had an effect [21]. Studies have demonstrated that moral efficacy and moral concern can mitigate the negative effects of toxic leadership on nurses for both perceivers and victims [22]. However, which characteristics of nurses will experience the most negative effects? Özkan et al. [8] concluded that nurses in Turkey who actively chose nursing as a career, who were willing to work in the current unit, who had higher levels of education, and who had received on-the-job training were less influenced by toxic managers.

Different outcomes may result from cultural differences between nations [23]. Under the influence of traditional culture, China is a collectivist society with a clear organizational hierarchy and a large power distance [24, 25]. Thus, there may be distinct cultural factors that contribute to toxic leadership in China. This study was conducted in Chinese hospitals to understand the status of toxic leadership behaviors, identify the relevant factors affecting nurses' perceptions. Understanding these factors may help hospital managers pay attention to the prevention of toxic leadership behaviors of nurse managers, help nurse managers optimize their own management behaviors, and help nurses recognize their own shortcomings, thus further reducing the incidence and adverse effects. This study provides a reference for countries around the world to understand toxic leadership behaviors and develop follow-up interventions to avoid negative effects.

## 2. Methods

### 2.1. Study Design, Setting, and Participants

According to the measures for the administration of the hospital grade in China, hospitals were divided into three levels, with the size of hospitals gradually increasing from primary hospital to tertiary hospital. Primary hospitals are community hospitals. Secondary hospitals are regional hospitals that provide medical and health services to multiple communities and undertake certain teaching and research tasks. Tertiary hospitals have comprehensive medical, teaching, and research capabilities and provide high-level specialist medical and health services to several regions. In this study, a convenience sampling method was used to select nurses from two tertiary hospitals and two secondary hospitals in Hubei, China.  Table 1 shows the details and sample allocation for each hospital. Nurses who provided patient direct care and volunteered to participate were selected, excluding nurses on internship and long-term leave (≥3 months). The sample size was estimated according to the Kendall method, and the sample size should be 5∼10 times more than the scale entries, considering that there may be 20% of invalid questionnaires, and the final sample size was 225∼450.

### 2.2. Measures

*Demographic Information Questionnaire*. It was used to collect personal information about the surveyed nurses, including gender, age, marital status, education, family location, hospital grade, department, years of experience, technical title, monthly income, employment mode, and night shift frequency. The questionnaire was designed by the researcher and added with night shift frequency items guided by expert opinion.

*Negative Behavior Scale for Nurse Managers*. The scale was developed by the investigators through a literature review and qualitative interviews, combined with the results of Delphi expert consultation and was primarily used to evaluate the toxic leadership behaviors of Chinese nurse managers [26]. The scale has 36 items divided into 6 dimensions: neglect of needs (7 items), personal attack (5 items), entitlement abuse (9 items), unpredictable behavior (4 items), slackness at work (6 items), and improper supervision (5 items). It covers various toxic leadership behaviors of nurse managers against subordinates, themselves, organizations and work. The questionnaire uses a 5-point Likert scale, with 1 to 5 indicating “never,” “occasionally,” “sometimes,” “usually,” and “always,” respectively. The higher the score, the more frequently the behavior occurred among nurse managers. The scale items gathered under six factors in the original scale explain 75.13% of the total variance. The previous study indicated that the reliability and validity of the scale were good, with a Cronbach's *α* of 0.880 and a content validity index of 0.930. In this study, the Cronbach's *α* was 0.925.

### 2.3. Data Collection

The study used an online questionnaire application (https://www.wjx.cn/) to collect data. Data collection started at the beginning of August 2022 and was finished by the end of October 2022. After obtaining permission from the nurse administrators at the identified hospitals, researchers directly distributed the online questionnaire links to nurses during their free time. Before distribution, the purpose, significance, content, precautions, and the principle of anonymity and voluntaries was explained. It can only be continued after study participants have given their consent, and they can withdraw at any time. Each ID account number can only be answered once, and only completed questionnaires can be submitted. Data from the platform were directly exported and entered using Excel 2019. The questionnaires were screened according to the exclusion criteria of the questionnaire, and abnormal questionnaires with too short (<1 min) or too long (>9 min) response times, answers in obvious patterns, or logical confusion were deleted.

### 2.4. Data Analysis

The data were analysed by SPSS 24.0 (IBM Corp.). The Shapiro–Wilk test concluded that the continuous data were all non-normal distribution, so the data was represented by the medians and quartiles (p25-p75). The Kruskal–Wallis *H* test or Mann–Whitney *U* test was used to test for differences between groups. The residual of the dependent variable with a skewed distribution satisfied linearity, independence, homogeneity of variance, and normal distribution, so multiple linear regression analysis can be performed to analyse the factors that affect nurses' perceptions of harmful leadership behaviors [27]. The total score of the toxic leadership behavior was set as the dependent variable, and all variables with statistically significant differences in the univariate analysis were entered into a multiple linear regression model as independent variables (*α* in = 0.05, *α* out = 0.10). All tests were two-sided and the level of acceptable significance was assumed to be 0.05.

### 2.5. Ethical Considerations

The study was approved by the Ethics Committee of Tongji Medical College of Huazhong University of Science and Technology (NO. S044). The whole protocol, in accordance with the ethical principles of the Helsinki Declaration and ensured the anonymity and confidentiality of the respondents.

## 3. Results

### 3.1. Relationship between Nurse Characteristics and Toxic Leadership Behavior

A total of 500 questionnaires were distributed, and 455 valid questionnaires were recovered, with a valid recovery rate of 91%. The average age of the respondents was 33.55 years (SD = 6.30), and the average years of work experience were 10.76 (SD = 6.62). Female (*n* = 423, 92.97%), married (*n* = 318, 69.89%), and bachelor's degree (*n* = 420, 92.30%) were predominant. There were significant differences in toxic leadership behaviors perceived by nurses with different gender, education, years of experience, departments, employment modes, and night shift frequency (all *P* < 0.05, Table 2).

### 3.2. Scores of Toxic Leadership Behaviors

Table 3 shows the overall score and average score for each dimension. The overall score of the negative leadership behavior scale was 109 (87, 123), and the average score of the item was 2.94 ± 0.92. Among all the dimensions, the score of improper supervision was the greatest (*M* = 3.00, SD = 0.86), and the score of personal attack was the lowest (*M* = 2.87, SD = 0.96).

### 3.3. Multiple Linear Stepwise Regression Analysis on Factors Influencing Nurses' Perception of Toxic Leadership Behavior

The multiple linear stepwise regression results showed that gender (female) ((*β* = 0.119, *P* = 0.001)), education(master's degree or above) (*β* = −0.089, *P* = 0.013), department (Pediatrics, Intensive Care Medicine) ((*β* = 0.161, *P* < 0.001); (*β* = 0.125, *P* < 0.001)), night shift frequency (5∼, ≥9) ((*β* = 0.447, *P* < 0.001); (*β* = 0.191, *P* < 0.001)) and employment mode (personnel agency, formal establishment) ((*β* = −0.123, *P* = 0.001); (*β* = 0.290, *P* < 0.001)) were significantly predicted nurses' perception of toxic leadership behavior, making it statistically significant (*R*^2^ = 0.441, adjusted *R*^2^ = 0.431, *F* = 43.922, *P* < 0.001), and explain a total of 43.1% of the variance of the dependent variable (Table 4).

## 4. Discussion

The average score for perceived abusive supervision is 2.94 ± 0.92, above the median of the questionnaire. This result indicates that nurses consider the negative leadership behavior of head nurses at a moderate level, which is consistent with a recent survey of 1240 nurses in Ghana [5], higher than the study by Labrague [12]. The highest scores are obtained for the dimension of improper supervision. This may be related to the nature of clinical nursing work, where nurses are short-staffed in a heavy and intense nursing workload, which requires high coordination and time management skills from the nurse managers. The lowest score on the dimension of personal aggression indicates that nurse managers pay more attention to the methods of communication with nurses in the workplace. It cannot be ruled out that, in the traditional context of China, the manager will conduct negative leadership in a more obscure way. There is a significant difference between male and female perceived negative leadership behavior scores of nurse managers, with female nurses' perceptions being higher, consistent with the findings of Li et al. [28]. Affective event theory points out that the evaluation of work events would be influenced by an individual's comprehensive understanding, feelings, and regulation of emotions [29]. Women's self-cognition mostly relies on external evaluation, while men generally acquire information from social comparison and self-cognition. In addition, compared with women, male nurses have stronger psychological endurance, more rigorous logical thinking, and problem-solving ability, which is a unique physical and psychological advantage [30] and enables them to be less affected by negative leadership in the work environment. This suggests that organizations should pay more attention to female nurses' psychological health. At the same time, female nurses need to strengthen their psychological resilience, clearly recognize themselves, and think rationally at work to avoid the influence of negative leadership behaviors of nurse managers.

Educational level is one of the factors that affect nurses' perceptions of negative leadership behaviors, with higher levels of education revealing less perceived. This is contrary to Özkan et al. [8] and Özer et al. [31] who concluded that nurses with graduate degrees were exposed to more toxic behaviors from their managers than other nurses. However, Han et al. [32] suggested that educational level can influence individual cognitive and psychosocial functioning, with higher levels of resilience among nurses with higher education. In this study, it is concluded that nurses with higher education have more professional theoretical level and clinical experience, have advantages in terms of career development and welfare benefits, have more initiative at work, can deal with problems more appropriately, and therefore have higher job satisfaction and less negative emotions. This suggests that managers should encourage nurses to constantly improve their educational level and increase their motivation for continuing education, thereby increasing their level of resilience. Organizations should also actively promote colleague support [33], provide group counseling training [34], and establish a social-level support system to reduce the negative impact of negative leadership behavior.

The department is one of the factors that influence nurses' perception of the negative leadership behavior of nurse managers. Different departments have differences in work intensity and personnel allocation. Patients in critical care units are in critical condition, and nursing staff is often under a high level of stress due to the intense mental and physical workload [35]. Similarly, pediatric nurses are usually faced with crying children and anxious parents, and pediatric care is more difficult [36]. Nurses in high-stress, high-intensity, and high-risk work environments are more sensitive to the perception of negative leadership behaviors and their more serious consequences [37]. The improvement of nurses' psychological capital level is beneficial to reducing the level of perceived stress [38]. Therefore, nursing managers should pay attention to the influence of work intensity and work environment on nurses' perceived negative leadership behaviors, actively guide the psychological situation and stress management of nurses working at high intensity, and take appropriate measures according to the characteristics of different nurses to avoid their psychological fluctuations as much as possible and stabilize the nursing team.

The nature of employment predicts nurses' perceived toxic leadership behaviors toward nurse managers. Nurses employed in personnel agencies or formal establishments reported less toxic leadership behaviors than in labor dispatch, which is consistent with the findings of Mehta and Maheshwari [39]. Because of the lack of job stability and benefits for nurses on dispatch, their job security is always uncertain, and therefore they are more sensitive to negative leadership behaviors. On the contrary, the nurses who have the establishment are mostly the hard core of the department, with higher professional titles, who can participate in or directly manage the affairs of the department. Their labor behavior and work are valued, and their interpersonal relationship handling ability and the sense of decent work are stronger [40]. The nature of employment can be regarded as an incentive. When subordinates are paid attention to and satisfied with the nature of employment, their job satisfaction and organizational commitment can be improved. Therefore, nurse managers need to strengthen their care and support for nurses in the labor dispatch and contract system, further promote and implement equal pay for equal work, and increase the overall economic income of nurses. Actively guiding nurses in their career development and reducing their perceived negative leadership behavior can also lead to a higher quality of care [41].

Night shift frequency is one of the influencing factors for nurses' perceptions of the negative leadership behaviors of nurse managers. Night shifts require intense mental and physical work, and long-term multifrequency night shifts could reduce nurses' sleep quality and disrupt normal life status. Then there would be a series of physiological and psychological changes, resulting in the production of negative emotions [42] and an increase the work pressure [43]. Negative emotions will narrow the range of personal attention and cognitive [44]. At the same time, if there is a load other than negative emotions, it will lead to a lack of personal cognitive resources and affect the perception of things and personal performance. Nurses with frequent night shift work have more negative emotions and heavy emotional regulation tasks, and they are more inclined to think that they are subjected to negative leadership behaviors. This suggests that nurses should strengthen their adaptability to night shift rotation, and nurse managers should rationally allocate human resources and create a quiet and comfortable rest environment for night shift nurses in the department. The circadian rhythm disorder of nurses with high night rotation frequency can be focused on through various physical examination means. If necessary, aromatherapy and other intervention methods can be used to ensure sleep quality [45].

### 4.1. Limitations

The following are the study's shortcomings. First, social expectation bias is a clear restriction because this study relied on nurses' self-reported data from an anonymous online questionnaire. Second, the participants were all from one city, which may affect the inference of the results. Third, toxic leadership behaviors are equally influenced by the nurse leaders and organizational management. However, we only identified nurse factors. Finally, this was a cross-sectional study, and causal relationships between variables could not be determined. Despite these limitations, this study provides valuable data that can be used to develop appropriate preventive strategies.

## 5. Conclusions

In summary, this study was conducted under the Helsinki Principles and the nurses perceived their nurse managers' toxic leadership behaviors to be moderate. Among the nurses, those who were female, had low levels of education, high unit assignments, unstable nature of employment, and a high number of monthly night shifts. They reported an increase in toxic leadership behaviors. These impact variables can be taken into consideration when developing interventions to protect nurses from or reduce the negative impact of toxic leadership behaviors of nurse leaders.

## 6. Implications for Nursing Management

Nurse managers should fully encourage nurses' initiative when arranging tasks and provide reasonable appeal channels. Hospital managers need to ensure a reasonable hierarchy of nursing staff in the department, as well as rationalize nursing tasks and appraisals to ensure that nurses are not overly distracted from their work. In addition, the personal characteristics of nurses also have an impact on the perception of negative leadership behavior of nurse managers. This requires nurses to enhance their psychological construction, actively improve their professional knowledge and skills, and pay attention to the improvement of their communication skills to eliminate negative leadership behaviors due to their own reasons. Nurses' perceptions of themselves are influenced by social identity, and organizational support can reduce the negative effects of negative leadership behaviors [46]. Organizations need to recognize and praise nurses' work contributions, guide nurses to change their perceptions and attitudes toward the nursing profession and nurse managers, and find reasonable ways to relieve nurses' stress and bad emotions to avoid the negative effects.

## Figures and Tables

**Table 1:** Detailed sampling allocation and response rate for each hospital.

Hospitals	Hospital level	Facility size	Total number of nurse managers	Total number of nurses
A	Tertiary	6905 beds	203	4102
B	Tertiary	3300 beds	110	1960
C	Secondary	630 beds	19	279
D	Secondary	578 beds	16	238

**Table 2:** Relationship between nurse characteristics and toxic leadership behavior (*n* = 455).

Characteristics	*N* (%)	*M* (p25, p75)	*z/H*	*P*
Gender^a^				
Male	32 (7.03)	91.00 (76.00, 112.25)	−3.025^*∗*^	0.002
Female	423 (92.97)	110.00 (88.00, 123.00)		
Age^b^				
<25	53 (11.65)	107.00 (82.50, 119.50)	6.111	0.191
25∼	103 (22.64)	107.00 (88.00, 120.00)		
30∼	140 (30.76)	112.00 (94.25, 126.00)		
35∼	105 (23.08)	109.00 (85.00, 121.50)		
≥40	54 (11.87)	106.50 (81.75, 119.75)		
Marital status^b^				
Married	318 (69.89)	109.50 (89.00, 123.00)	1.221	0.543
Unmarried	130 (28.57)	109.00 (84.00, 120.00)		
Other^①^	7 (1.54)	111.00 (105.00, 135.00)		
Education^b^				
College degree or below	21 (4.62)	113.00 (87.00, 127.00)	12.710^*∗*^	0.002
Bachelor's degree	420 (92.30)	110.00 (88.25, 123.00)		
Master's degree or above	14 (3.08)	81.00 (75.50, 85.25)		
Family location^a^				
Local	201 (44.18)	111.00 (86.00, 123.50)	−0.351	0.725
Nonlocal	254 (55.82)	108.00 (88.75, 122.25)		
Years of experience^b^				
<5	112 (24.62)	107.00 (84.00, 119.00)	13.387^*∗*^	0.010
5∼	110 (24.18)	112.50 (96.00, 125.25)		
10∼	108 (23.73)	110.00 (91.00, 124.00)		
15∼	85 (18.68)	113.00 (85.00, 125.00)		
≥20	40 (8.79)	102.00 (76.25, 115.75)		
Department^b^				
Internal medicine	110 (24.18)	109.00 (86.75, 119.00)	36.178^*∗∗*^	<0.001
Surgery	108 (23.74)	107.00 (84.50, 118.75)		
Obstetrics and gynecology	55 (12.09)	101.00 (79.00, 120.00)		
Pediatrics	50 (10.99)	121.00 (108.00, 134.00)		
Operating room	67 (14.73)	107.00 (82.00, 125.00)		
Intensive care medicine	38 (8.35)	119.50 (107.00, 133.25)		
Other^②^	27 (5.92)	106.00 (89.00, 112.00)		
Hospital grade^a^				
Secondary hospital	189 (41.54)	108.00 (86.00, 120.50)	−1.102	0.270
Tertiary hospital	266 (58.46)	110.00 (88.00, 124.25)		
Employment mode^b^				
Labor dispatch	37 (8.13)	122.00 (85.25, 136.75)	48.236^*∗∗*^	<0.001
Contract system	340 (74.73)	112.00 (94.00, 124.00)		
Personnel agency	40 (8.79)	80.00 (59.50, 108.50)		
Formal establishment	38 (8.35)	99.50 (81.00, 114.75)		
Technical title^b^				
Junior nurse	76 (16.70)	107.00 (85.50, 116.00)	5.825	0.120
Junior nurse practitioner	193 (42.42)	111.00 (88.50, 125.00)		
Supervising nurse practitioner	179 (39.34)	111.00 (84.00, 124.00)		
Associate chief nurse practitioner	7 (1.54)	106.00 (104.00, 108.00)		
Night shift frequency (month)^b^				
0	55 (12.09)	84.00 (69.00, 106.00)	120.939^*∗∗*^	<0.001
1∼	96 (21.10)	88.00 (73.50, 107.00)		
5∼	279 (61.32)	117.00 (103.00, 128.00)		
≥9	25 (5.49)	112.00 (107.50, 118.00)		
Monthly income^b^				
<5000	47 (10.33)	109.00 (87.00, 125.00)	6.026	0.110
5000∼	139 (30.55)	110.00 (89.00, 122.00)		
10000∼	183 (40.22)	111.00 (92.00, 125.00)		
≥15000	86 (18.90)	102.50 (81.00, 116.50)		

^a^Mann–Whitney *U* test. ^b^Kruskal–Wallis *H* test. ^*∗*^*P* < 0.01. ^*∗∗*^*P* < 0.001. ^①^Divorced or widowed. ^②^Including outpatient department, interventional radiology, general medicine, rheumatology, dermatology, and traditional Chinese medicine.

**Table 3:** Scores of toxic leadership behaviors (*n* = 455).

Dimensions	Total score *M*(P_25_, P_75_)	Average item score ($\bar{x}$ ± SD)
Neglect of needs	20 (14, 26)	2.94 ± 0.94
Personal attack	14 (10, 18)	2.87 ± 0.96
Entitlement abuse	27 (21, 34)	2.93 ± 0.81
Unpredictable behavior	11 (9, 15)	2.93 ± 0.93
Slackness at work	18 (14, 22)	2.90 ± 0.85
Improper supervision	15 (12, 19)	3.00 ± 0.86

**Table 4:** Multiple linear stepwise regression analysis on factors influencing nurses' perception of toxic leadership behavior (*n* = 455).

Variables	B (95% CI)	*SE*	*β*	*t*	*P*
(Constant)	81.895 (75.506, 88.284)	3.251		25.192	<0.001
Gender: *R* = male					
Female	10.587 (4.398, 16.776)	3.149	0.119	3.362	0.001
Education: *R* = college degree or below					
Master's degree or above	−11.736 (−20.967, −2.506)	4.697	−0.089	−2.499	0.013
Department: *R* = Internal medicine					
Pediatrics	11.363 (6.372, 16.355)	2.540	0.161	4.474	<0.001
Intensive care medicine	10.360 (4.423, 16.298)	3.021	0.125	3.429	0.001
Night shift frequency (month): *R* = 0					
5∼	22.216 (18.729, 25.703)	1.774	0.447	12.521	<0.001
≥9	18.967 (11.655, 26.278)	3.720	0.191	5.098	<0.001
Employment mode: *R* = labor dispatch					
Personnel agency	−10.058 (−15.827, −4.290)	2.935	−0.123	−3.472	0.001
Formal establishment	−24.080 (−29.985, −18.174)	3.005	−0.290	−8.014	<0.001

## Data Availability

The data that support the findings of this study are available from the corresponding author upon reasonable request.
